# Guillain-Barré Syndrome-Related *Campylobacter jejuni* in Bangladesh: Ganglioside Mimicry and Cross-Reactive Antibodies

**DOI:** 10.1371/journal.pone.0043976

**Published:** 2012-08-27

**Authors:** Zhahirul Islam, Michel Gilbert, Quazi D. Mohammad, Kevin Klaij, Jianjun Li, Wouter van Rijs, Anne P. Tio-Gillen, Kaisar A. Talukder, Hugh J. Willison, Alex van Belkum, Hubert P. Endtz, Bart C. Jacobs

**Affiliations:** 1 Emerging Diseases and Immunobiology, Centre for Food and Waterborne Diseases, icddr,b, Dhaka, Bangladesh; 2 Department of Medical Microbiology and Infectious Diseases, Erasmus MC, University Medical Centre, Rotterdam, The Netherlands; 3 National Research Council Canada, Ottawa, Ontario, Canada; 4 Department of Neurology, Dhaka Medical College Hospital, Dhaka, Bangladesh; 5 Neurology and Immunology, Erasmus MC, University Medical Centre, Rotterdam, The Netherlands; 6 College of Medical, Veterinary and Life Sciences, University of Glasgow, Glasgow, United Kingdom; Charité-University Medicine Berlin, Germany

## Abstract

**Background:**

*Campylobacter jejuni* is the predominant antecedent infection in Guillain-Barré syndrome (GBS). Molecular mimicry and cross-reactive immune responses to *C. jejuni* lipo-oligosaccharides (LOS) precipitate the development of GBS, although this mechanism has not been established in patients from developing countries. We determined the carbohydrate mimicry between *C. jejuni* LOS and gangliosides, and the cross-reactive antibody response in patients with GBS in Bangladesh.

**Methodology:**

Sera from 97 GBS patients, and 120 neurological and family controls were tested for antibody reactivity against LOS from *C. jejuni* isolates from GBS patients in Bangladesh (BD-07, BD-39, BD-10, BD-67 and BD-94) by enzyme-linked immunosorbent assay (ELISA). Cross-reactivity to LOS was determined by ELISA. The LOS outer core structures of *C. jejuni* strains associated with GBS/MFS were determined by mass spectrometry.

**Principle Findings:**

IgG antibodies to LOS from *C. jejuni* BD-07, BD-39, BD-10, and BD-67 IgG antibodies were found in serum from 56%, 58%, 14% and 15% of GBS patients respectively, as compared to very low frequency (<3%) in controls (*p*<0.001). Monoclonal antibodies specific for GM1 and GD1a reacted strongly with LOS from the *C. jejuni* strains (BD-07 and BD-39). Mass spectrometry analysis confirmed the presence of GM1 and GD1a carbohydrate mimics in the LOS from *C. jejuni* BD-07 and BD-39. Both BD-10 and BD-67 express the same LOS outer core, which appears to be a novel structure displaying GA2 and GD3 mimicry. Up to 90–100% of serum reactivity to gangliosides in two patients (DK-07 and DK-39) was inhibited by 50 µg/ml of LOS from the autologous *C. jejuni* isolates. However, patient DK-07 developed an anti-GD1a immune response while patient DK-39 developed an anti-GM1 immune response.

**Conclusion:**

Carbohydrate mimicry between *C. jejuni* LOS and gangliosides, and cross-reactive serum antibody precipitate the majority of GBS cases in Bangladesh.

## Introduction

Guillain-Barré syndrome (GBS) is an acute post-infectious immune-mediated peripheral neuropathy with a marked variation in pathology, clinical presentation and prognosis [1]. Although poliomyelitis has been eradicated in Bangladesh, non-polio acute flaccid paralysis (AFP) cases are still frequently diagnosed. The majority of the non-polio AFP cases are diagnosed as GBS [2]. The crude incidence rates of GBS among children <15 years of age varied from 1.5 to 1.7 per 100,000 per year in Bangladesh [2]. This crude incidence rate of GBS appeared to be 2.5 to 4 times higher than that other parts of the world [2]. *Campylobacter jejuni* is recognized as the most common pathogen associated with GBS and Miller Fisher syndrome (MFS) [3], [4], [5], [6].

The exact pathogenesis of post-*Campylobacter* neuropathy such as GBS is not clearly understood, however, molecular mimicry between *Campylobacter* lipo-oligosaccharides (LOS) and gangliosides in nervous tissue induces a cross-reactive antibody response [7], [8]. Antibody reactivity against GM1, GM1b, and GalNAc-GD1a is associated with pure motor GBS [9], and anti-GQ1b antibody reactivity has a strong association with oculomotor symptoms and ataxia [10]. The oligosaccharide core of LOS molecules expressed by *C. jejuni* structurally resemble the oligosaccharide core of certain molecules present in neural tissue [11], [12]. Many studies have been carried out in the developed world to establish the pathogenesis of *C. jejuni*-induced GBS but little information is available concerning the pathogenic mechanism in neuropathy-associated strains from developing countries such as Bangladesh.

Recently, we reported an unusually high frequency of acute motor axonal neuropathy (AMAN) variant of GBS in Bangladesh, associated with preceding *C. jejuni* infections and the presence of serum antibodies against GD1a and GM1 [6]. Unfortunately, for many patients in Bangladesh the current standard treatment for GBS are too expensive. To develop more effective and targeted therapies, improved understanding of GBS pathogenesis is required. The aim of the present study was to investigate the role of molecular mimicry and cross-reactive IgG responses in GBS in Bangladesh. The LOS outer core of *C. jejuni* strains isolated from these patients was characterized for the presence of ganglioside like structures. In addition, sera from patients with GBS and controls were screened for antibodies to *C. jejuni* LOS and the cross-reactivity to gangliosides.

**Table 1:** Clinical and laboratory findings in five patients with Guillain-Barré syndrome from whom *Campylobacter jejuni* was isolated and used in the current study^a^.

Patients	BD-39	BD-07	BD-10	BD-67	BD-94
**Clinical characteristics**					
Age (years)	30	40	15	40	10
Sex	F	M	M	M	F
Diarrhoea	+	+	+	+	–
Days to nadir	12	2	5	6	3
Ophthalmoplegia	–	–	+	+	–
Sensory deficits	–	–	–	–	–
Motor deficits	+	+	+	+	+
**GBS disability score**					
At nadir	5	4	5	4	5
At 26 weeks	4	0	3	3	4
**Serology**					
Anti-GM1 IgG	+	–	–	+	–
Anti-GD1a IgG	–	+	+	+	–
Anti-GQ1b IgG	–	–	+	+	–
*C. jejuni* serology	+	+	+	+	+
***C. jejuni*** isolates					
Penner serotype	HS:19	HS:19	HS:23	HS:23	HS:21

a. Abbreviations: M, Male; F, Female; +, present; -, absent; N, normal, GBS disability score (6).

## Materials and Methods

### Patients and Controls

In this study 100 consecutive patients with GBS or MFS were admitted to Dhaka Medical College Hospital (DMCH), Bangabandhu Sheikh Mujib Medical University (BSMMU) and Dhaka Central Hospital (DCH) between July 2006 and June 2007 [6]. All patients fulfilled the diagnostic criteria for GBS [13], as evaluated by a neurologist and a senior neurologist [6]. Data were collected prospectively on age, sex, antecedent events, detailed neurological signs and symptoms, treatment, days to nadir, complications, duration of admission and clinical disease severity (expressed for weakness as Medical Research Council (MRC) sum score and for disability as the GBS disability score measured at entry. Two types of controls were selected for patients: the first control was a family member living in the same household (family control, FC); the second control was an age and sex matched patient hospitalized in the same ward with other neurological disease (OND). Blood and up to three stool samples were collected from all patients and controls. All studies were approved by the ethical committee of Dhaka Medical College, Dhaka and all patients gave written informed consent [6].

### *C. jejuni* and Lipo-oligosaccharides

*C. jejuni* was isolated from stool specimens of 10 patients with GBS or MFS [14]. Five of these *C. jejuni* isolates were selected for serological studies and definition of the molecular mimicry. The clinical features and laboratory findings of these patients are given in Table 1. The *C. jejuni* isolates, BD-07, BD-10, BD-39, BD-67 and BD-94 [14], were classified according to the heat-stable (HS) serotyping system developed by Penner [15]. The LOS fraction from all *C. jejuni* strains was isolated by hot phenol-water extraction and processed as described before [16], [17]. LOS from the *C. jejuni* serostrain HS:03 (CCUG 10937), lacking gangliosides mimicry [11], was included for control studies.

### Antibody Reactivity to *C. jejuni* LOS

Pre-treatment, acute phase serum samples from 97 of these 100 GBS patients, including the 5 patients from whom *C. jejuni* was isolated, were available for serological studies. Serum samples from 60 family controls (FC) and 60 controls with other neurological disease controls (OND) were analysed. The sera were tested for IgG, IgM and IgA activity against LOS from *C. jejuni* (BD-07, BD-10, BD-39, BD-67, BD-94 and CCUG 10937) in ELISA. Serum antibodies against LOS were tested in ELISA as described earlier [18], [19] with some modifications. In brief, a 96-well polystyrene microtitre trays (Immuno Maxisorb, Nunc) was coated with 1 µg of LOS in 50 µl PBS (pH = 7.8) per well, and incubated overnight at 37°C. Non-specific binding sites were blocked with PBS containing 1% BSA (Sigma) for 2 hours at room temperature, and for another 2 hours at 4°C. After blocking, the plates were incubated overnight at 4°C with serum diluted 1∶1000 in PBS–1% BSA. After washing with PBS (pH = 7.8), the plates were incubated with peroxidase-conjugated rabbit anti-human IgG, IgA and IgM antibody (Sanbio) diluted 1∶2500 in PBS-1% BSA, for 90 min at room temperature. After washing with PBS, the plates were developed with *O*-phenyl diamine (Sigma) in citrate buffer (pH 5.1) and the optical densities (ODs) were read at 492 nm. Each individual serum sample was tested in triplicate, this test means that each serum sample was tested in 3 LOS-coated wells and 3 blank wells in the same ELISA. The mean absorbance value for triplicate reference wells without antigen was subtracted from the mean value for triplicate sample wells with the antigen. A serum was considered positive for anti-LOS reactivity when the corrected OD was higher than the mean value of controls plus 3 times standard deviation (SD). All serological studies were performed blinded for clinical data.

### Antibody Reactivity to Gangliosides

Pre-treatment serum samples obtained upon hospitalization were available from 97 of these 100 patients and 120 controls to determine the presence of IgM and IgG antibodies to the gangliosides GM1, GD1a and GQ1b by ELISA according to methods and criteria for positivity previously described [20].

### Determination of Cross-reactivity

Cross-reactivity of anti-ganglioside antibodies to *C. jejuni* LOS was determined by pre-incubation of serum with LOS from the *C. jejuni* isolated from the autologous patients and with LOS from the *C. jejuni* serostrain HS:03 (CCUG 10937) as a control, according to methods previously described [17]. To detect cross-reactive antibodies, serum DK-07 (diluted 1∶100) and DK-39 (diluted 1∶100) were pre-incubated with LOS from their autologous *C. jejuni* isolate and as a control with LOS from the *C. jejuni* serostrain HS:03 (CCUG 10937). *C. jejuni* LOS concentrations of 200, 50, 12.5, 3.1, 0.8 and 0.2 µg/ml were incubated with serum for 3 hours at 4°C. After the incubation with LOS, the sera were centrifuged at 3000 rpm for 5 minutes at 4°C. The supernatants were tested for residual anti-ganglioside IgG reactivity by ELISA. The percentage of inhibition was defined as:
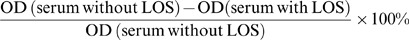



### Mass Spectrometry Analysis

LOS fractions from *C. jejuni* BD-07, BD-10, BD-39, BD-67 and BD-94 were prepared for mass spectrometric analysis. These *C. jejuni* strains were grown overnight at 37°C on Butzler agar plates in a microaerobic atmosphere. Material from one confluent agar plate was harvested and treated with proteinase K at 60 µg/ml, RNase A at 200 µg/ml, and DNase I at 100 µg/ml (Promega, Leiden, The Netherlands). *O*-deacylated LOS samples were prepared and analyzed by capillary electrophoresis coupled to electrospray ionization mass spectrometry (CE-ESI-MS) [21].

### Statistical Analysis

Differences in median values were tested with the Mann-Whitney *U* test. Differences in proportions were tested with the chi square test or Fisher’s exact test. Differences were considered significant at p<0.05 after two-sided testing. Statistical analysis was performed using InStat version 4.0 (Graphpad Software, San Diego, CA).

## Results

### Antibodies to *C. jejuni* LOS

**Figure 1:**
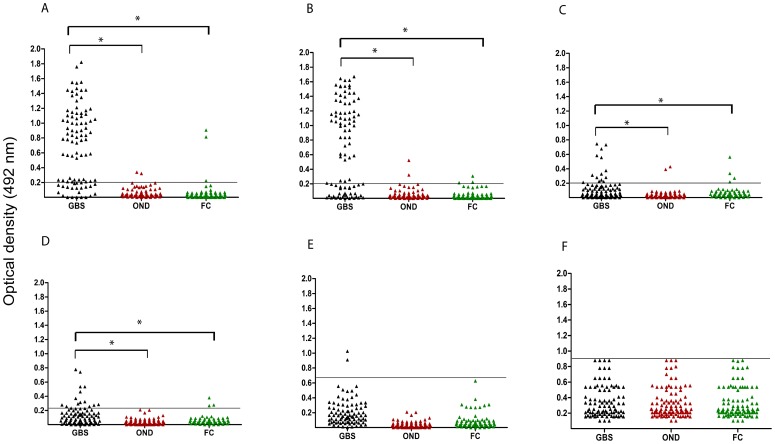
Serum samples from patients with GBS and from controls with other neurological disease (OND) and family controls (FC) were tested for IgG activity to lipo-oligosaccharides (LOS) from 6 *C. jejuni*. Subcharts represent serum IgG activity to LOS from (**A**) *C. jejuni* BD-07 isolated from a GBS patient (**B**) *C. jejuni* BD-39 isolated from a GBS patient, (**C**) *C. jejuni* BD-10 isolated from a GBS/MFS patient, (**D**) *C. jejuni* BD-67 isolated from a GBS/MFS patient, (**E**) *C. jejuni* BD-94 isolated from a GBS patient (**F**) *C. jejuni* Penner HS:03 serostrain (CCUG 10937), lacking ganglioside mimics, as a control. *p<0.001. Lines () indicate cut-off value. GBS, Guillain-Barré syndrome; OND, other neurological disease; FC, family controls; LOS, lipo-oligosaccharides.

The IgG reactivity in serum from the patients with GBS, and the FC and OND control groups to *C. jejuni* LOS is shown in Figure 1. IgG antibodies to LOS from BD-07, BD-39, BD-10, and BD-67 IgG were found in sera from 56%, 58%, 14% and 15% of GBS patients respectively, compared to very low frequencies (<3%) of controls (all *p*<0.001). Except for three cases, no sera from GBS patients and controls were positive for anti-LOS BD-94 IgG. The IgG activity to LOS from the control *C. jejuni* HS:03 serostrain CCUG 10937 in serum from the GBS patients did not differ from that in the two control groups. Serum IgA antibodies to LOS from BD-07 and BD-39 were found in 50% and 48% of GBS patients, respectively, compared 0% in OND and 4% in FC in controls (p<0.001) (data not shown). Serum IgM antibodies to LOS from BD-07 were found in 22% of GBS patients, compared to 4% of OND controls (*p* = 0.052) and 0% of family controls (*p* = 0.012) (data not shown). Serum IgM antibodies to LOS from BD-39 antibodies were found in 20% of GBS patients, in 4% of OND controls (*p* = 0.088) and in 0% of family controls (*p* = 0.025) (data not shown). In addition, diarrhea in the four weeks preceding GBS was reported in 26 (48%) of the 54 patients with IgG to *C. jejuni* LOS compared to 10 (23%) of 43 of the without anti-LOS patients (P = 0.02).

### Antibodies to Gangliosides

Serum antibodies to the ganglioside GM1, GD1a and GQ1b were more frequent in GBS patients (56%) compared to OND (1%) and FC (6%), p<0.001) [6]. The GBS patients were divided into two subgroups according to their anti-ganglioside reactivity: one group with anti-GM1 and/or anti-GD1a antibodies and another group without these antibodies. Anti-LOS reactivity was significantly associated with the presence of anti-ganglioside antibodies (p<0.001) (Table 2). Diarrhea in the four weeks preceding GBS was reported in 26 (48%) of the 54 ganglioside-positive patients, which was more frequent compared to the 10 (19%) of 43 of the ganglioside-negative patients (P<0.01) (Table 2). There was no association between anti-GM1/GD1a serology and sex, age, degree of overall disability of arms, facial, and bulbar weakness at the peak of the illness.

**Table 2:** Clinical characteristics of GBS patients associated with presence IgM and/or IgG antibodies in serum to the gangliosides GM1, GD1a and GQ1b*.

	Serum IgM and/or IgG antibodies to GM1, GD1a and/or GQ1b			
	Positive (N = 54)	Negative (N = 43)	Odds ratio (95% CI)	P-value†
**Clinical features**				
Age (median)	18 (2–52)	21 (5–65)	–	n.s.
Sex	41M/13F	29M/14F	1.07 (0.4–2.86)	n.s.
Preceding diarrhea	26 (48%)	10 (19%)	3.06 (1.6–8.2)	<0.02
Ventilation	10 (18%)	13 (30%)	0.56 (0.2–1.5)	n.s.
Sensory deficit at entry	0 (0%)	8 (14%)	–	0.001
***C. jejuni*** ** infections**				
Positive *C. jejuni* serology	43 (80%)	12 (28%)	10.1 (3.6–29.1)	<0.001
Positive anti-LOS serology	47 (87%)	10 (23%)	22.1 (6.9–75.5)	<0.001
**Electrophysiology (N = 64)**				
AMAN, AMSAN‡	28/42 (67%)	15/22 (68%)	0.9 (0.3–3.2)	n.s.
AIDP§	8/42 (19%)	6/22 (27%)	0.8 (0.5–1.4)	n.s.
Unclassified	6/42 (14%)	1/22 (5%)	3.5 (0.4–82.5)	n.s.

* Data were expressed as median or number of patients (percentage); M, male; F, female; CI, confidence interval; – cannot be calculated.

† Determined by Chi-square or Fisher’s exact test.

¶ Determined by Wilcoxon-Mann-Whitney *U* test.

‡ Axonal variants: acute motor axonal neuropathy (AMAN), acute motor sensory axonal neuropathy (AMSAN).

§ AIDP, acute inflammatory demyelinating polyneuropathy.

### Monoclonal Anti-ganglioside Antibodies Cross-reacting with *C. jejuni* LOS

**Figure 2:**
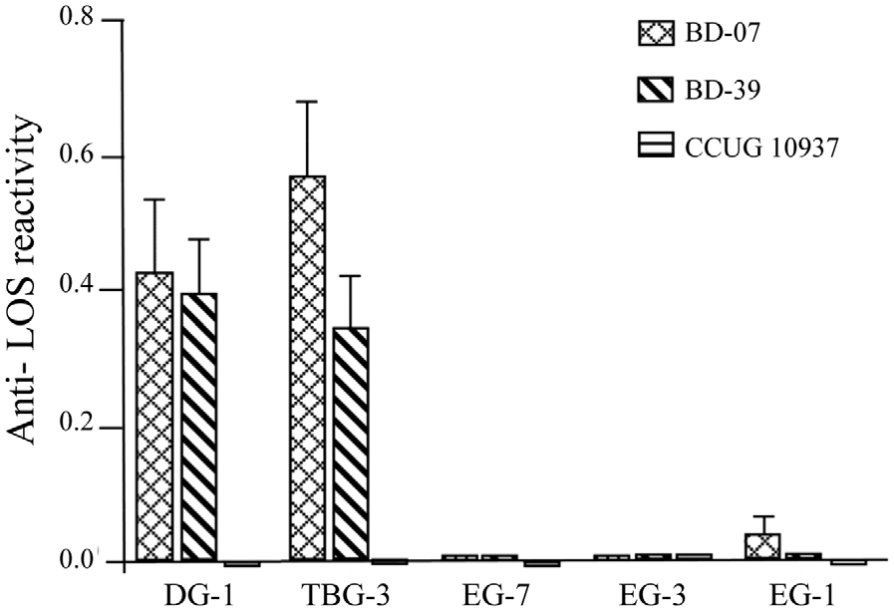
Binding of mouse monoclonal antibodies against LOS from *C. jejuni* BD-07 and BD-39. DG-1 (binding to GM1), TBG-3 (binding to GD1a), EG-7 (binding to GD1b), EG-3 (binding to GQ1b) and EG-1 (binding to GQ1b/GT1a).

Monoclonal mouse antibodies against different gangliosides were used to determine if ganglioside-mimics were present in the LOS. Monoclonal antibodies DG-1 (binding to GM1), and TBG-3 (binding to GD1a) reacted strongly with LOS from the *C. jejuni* strains (BD-07 and BD-39 respectively) as shown in Figure 2. These serological data indicate that *C. jejuni* BD-07 and BD-39 both have a GM1 and/or GD1a mimicking LOS. Control studies with monoclonal antibodies EG-7 (binding to GD1b), EG-3 (binding to GQ1b/GT1a) and EG-1 (binding to GQ1b) did not bind to the LOS, demonstrating that BD-07 and BD-39 have no GD1b, GQ1b or GQ1b/GT1a mimicry. The monoclonal antibodies bound to positive control *C. jejuni* LOS with known ganglioside-mimics (data not shown). None of the antibodies were bound by the LOS from either *C. jejuni* CCUG 10937 (negative control) or from BD-10, BD-67 and BD-94.

### Cross-reactive Antibodies to *C. jejuni* LOS and Gangliosides in GBS Patients

**Figure 3:**
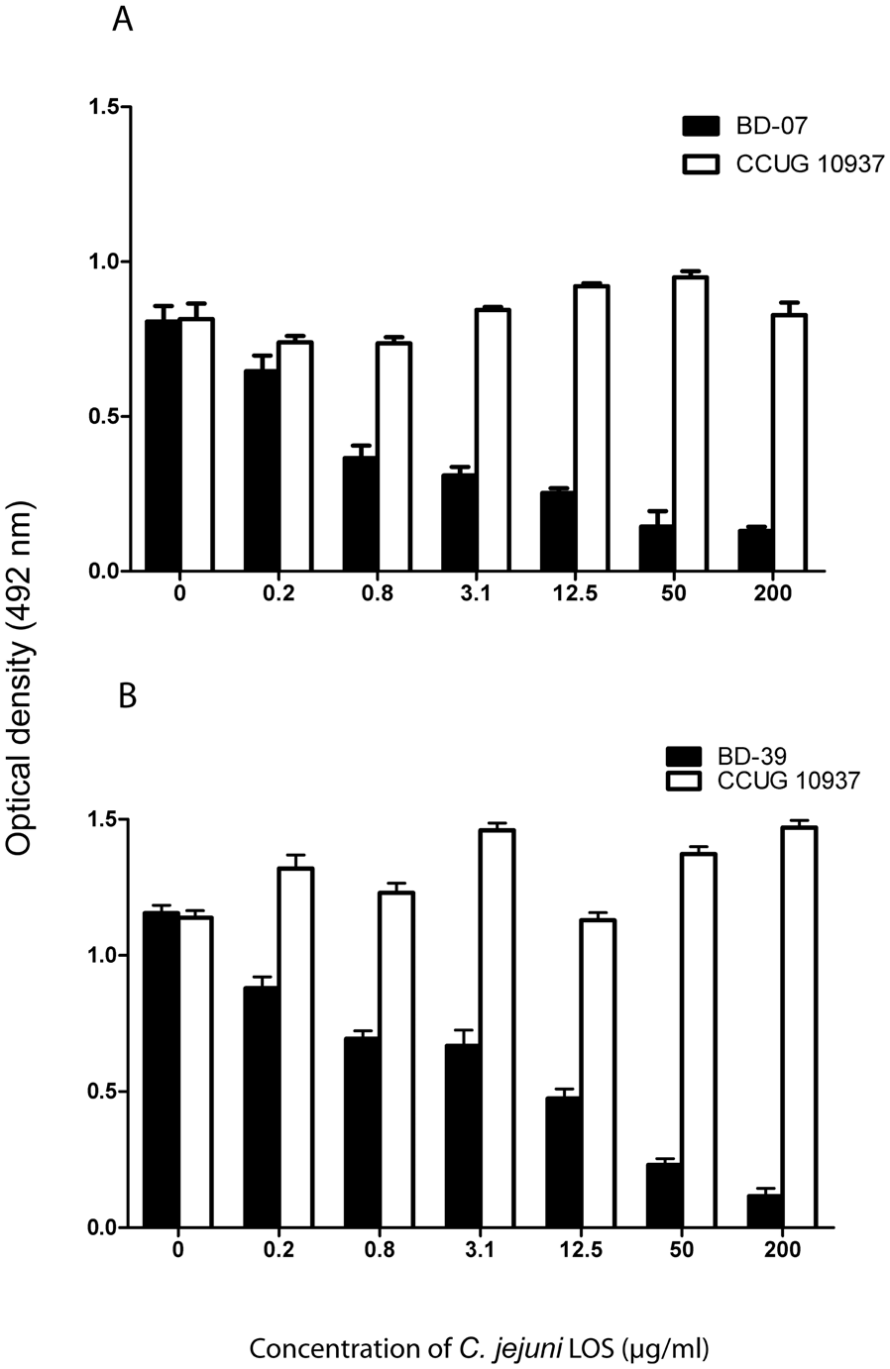
ELISA analysis of the cross-reactivity of GBS patient’s serum anti-ganglioside antibodies to LOS from the autologous *C. jejuni* strains. **A)** Optical density (OD) of IgG anti-GD1a reactivity in serum from patient DK-07 by pre-incubation with LOS from the autologous *C. jejuni* BD-07 strain (GM1/GD1a mimic, Table 3) and from Penner HS:03 serostrain (CCUG 10937). **B)** Optical density (OD) of IgG anti-GM1 reactivity in serum from patient DK-39 by pre-incubation with LOS from the autologous *C. jejuni* BD-39 strain (GM1/GD1a mimic, Table 3) and from Penner HS:03 serostrain (CCUG 10937). Graphs represent data as mean ± standard error.

Serum DK-07 and serum DK-39 showed reduced anti-ganglioside IgG antibody reactivity after pre-incubation with LOS from their autologous *C. jejuni* isolates (Figure 3). Serum DK-07 was tested for residual anti-GD1a IgG antibodies, whereas serum DK-39 was tested for anti-GM1 IgG antibodies. The percentage of inhibition of anti-ganglioside reactivity was dose-dependent with the LOS concentration. About 50% of inhibition was seen after pre-incubation with 3.1 µg/ml of LOS BD-07 and with 12.5 µg/ml of LOS BD-39. In control studies, LOS from *C. jejuni* Penner HS:03 serostrain (CCUG 10937) did not inhibit the ganglioside antibodies of serum DK-07 and DK-39, indicating that the cross-reactive anti-ganglioside antibodies showed no specific binding to the LOS from *C. jejuni* CCUG 10937 (Figure S1).

### Ganglioside Mimicry of *C. jejuni* LOS

**Figure 4:**
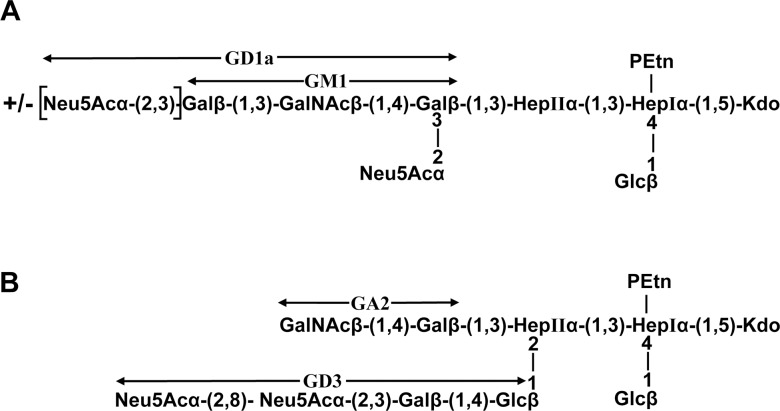
Proposed LOS outer core structures based on capillary-electrophoresis electrospray ionization mass spectrometry analysis of *O*-deacylated LOS samples (see Table S1). (A) strains BD-07 and BD-39 show mimicry with GM1 and GD1a; (B) strains BD-10 and BD-67 show mimicry with GA2 and GD3. Terminal regions mimicking gangliosides are indicated with arrows.

CE-ESI-MS of *O*-deacylated LOS was used to define the LOS outer core structures from five *C. jejuni* strains (Figure 4 and Table S1). The CE-ESI-MS did not provide linkage information but provided information about the sugar composition of the LOS outer core. The glycosyltransferase variants present in the LOS locus of each strain (Table 3) and the comparison with strains of known LOS outer core structures were used to propose LOS outer core structures for four strains (BD-07, BD-10, BD-39 and BD-67). The CE-ESI-MS data were not sufficient to propose a structure in the case of BD-94. However, we can conclude that the LOS outer core of BD-94 does not show ganglioside mimicry because no major ion was observed in a precursor ion mass spectrum at *m/z* 290.2 (data not shown), confirming that there is no sialic acid present. The CE-ESI-MS data obtained with BD-07 and BD-39 showed mass species with either one or two sialic acids (Table S1) which are proposed to be derived from GM1 and GD1a mimicry (Figure 4). Triple charged ion at *m/z* 1289.3 revealed a fragment ion at *m/z* 290.2 (NeuAc) and none at 581.3 (NeuAc-NeuAc). Since the composition of the triply charged ion at m/z 1289.3 contains two NeuAc, we conclude that these two residues are present on different Gal residues, which is consistent with GD1a mimicry. The glycosyltransferase variants in the LOS biosynthesis locus of BD-07 and BD-39 are consistent with GM1/GD1a mimicry (Table 3). They both contain a single domain glucosyltransferase (Cj1135) and an active β-1,3-galactosyltransferase (Cj1136) which suggests that the inner core will be extended with a Galβ-β1,3-linked residue on HepII. The Gal residue will be modified with a single NeuAc since the Cst-II variant in BD-07 and BD-39 is mono-functional (Thr51). The outer core is further extended with GalNAc and Gal residues by variants of CgtA and CgtB, respectively, which are specific for sialylated acceptors. The terminal Gal residue is partially substituted by the mono-functional Cst-II, which results in a mixture of GM1 and GD1a mimicry.

**Table 3:** Variants of the glycosyltransferases involved in synthesis of LOS outer core structures in *C. jejuni* strains BD-07, BD-10, BD-39 and BD-67^a^.

Strains	LOS class	GeneBank Accession No.	Cj1135	Cj1136	CgtAI	CgtAII	CgtB	Cst-II	GalT^g^
BD-07	A	GU289927	One-domain	On^b^	Mono-sialyl.^c^	Absent	Mono-sialyl.	Mono-f	Absent
BD-10	B	GQ249164	Two-domain	On	Non-sialyl.^d^	Off^e^	Off	Bi-	Present
BD-39	A	GU289928	One-domain	On	Mono-sialyl.	Absent	Mono-sialyl.	Mono-	Absent
BD-67	B	GQ249165	Two-domain	On	Non-sialyl.	Off	Off	Bi-	Present

a Assignment of the glycosyltransferase variants is based on amino acid sequence comparisons with variants of known specificities.

b on: indicates that a gene has no frame-shift mutation.

c Mono-sialyl.: the glycosyltransferase is specific for mono-sialylated acceptors.

d Non-sialyl.: the glycosyltransferase is specific for non-sialylated acceptors.

e off: indicates that a gene is inactive because of a frame-shift mutation.

f Cst-II variants: mono-: monofunctional, Cst-II has α-2,3-sialyltransferase activity. bi-: bifunctional, Cst-II has both α-2,3-sialyltransferase and α-2,8-sialyltransferase activity.

g GalT: β-1,4-galactosyltransferase that uses Glcβ-1,2-HepII- as acceptor.

CE-ESI-MS of *O*-deacylated LOS from strains BD-10 and BD-67 resulted in spectra with similar mass species (Table S1). These two strains also have LOS biosynthesis loci that are 100% identical to each other (GenBank accession numbers GQ249164 and GQ249165). We thus propose that they both express the same LOS outer core, which appears to be a novel structure with two extension sites from the inner core and two branches mimicking GA2 and GD3, respectively (Figure 4). The presence of di-NeuAc on one of the branches is confirmed by the fragment ion at *m/z* 581.3 when tandem mass spectrometry was carried out on the triply charged ion at *m/z* 1302.3 (data not shown). The Cst-II variant in these two strains is bi-functional (Asn51) which further supports the presence of di-NeuAc. The absence of a terminal Galβ-1,3-linked residue is consistent with a *cgtB* gene that has a frame-shift mutation.

## Discussion

In Bangladesh, preceding infections with *C. jejuni* are identified in the majority of patients with GBS [6]. In the current study we provide evidence for the hypothesis that *C. jejuni* infections induce GBS in these patients by molecular mimicry and induction of a cross-reactive immune response to nerve ganglisoides. This hypothesis is supported by our findings that [1] the serum IgG response to *C. jejuni* LOS and to gangliosides are closely associated in patients with GBS [2], patient serum anti-ganglioside IgG antibodies cross-react to *C. jejuni* LOS [3], mouse monoclonal anti-ganglioside antibodies cross-react to *C. jejuni* LOS [4], the *C. jejuni* isolates from Bangladeshi GBS patients have a LOS biosynthesis class A associated with ganglioside mimicry [5], and mass spectrometry analysis of LOS from *C. jejuni* isolates from Bangladeshi GBS patients demonstrated glycan structures that are identical to those of gangliosides. To our knowledge, this is the first report in which mass spectrometry is combined with DNA sequence data to determine the LOS outer core structures of neuropathy-associated *C. jejuni* strains isolated in South Asia. Our data confirm that ganglioside mimicry is the most likely pathogenic mechanism in the majority of *C. jejuni*-associated GBS cases. Our data further support the hypothesis that antecedent *C. jejuni* infections in GBS trigger the production of antibodies that cross-react with gangliosides.

Molecular mimicry between gangliosides and LOS has been demonstrated with *C. jejuni* isolates from GBS and MFS patients [22], [23], [24], [25], [26], [27]. Several findings in the current study support the hypothesis that cross-reactive antibodies to ganglioside in these patients contributed to the development of GBS in Bangladesh. Anti-LOS antibody levels are significantly associated with GBS as compared with data from two control groups (p<0.001). We provide strong evidence that anti-LOS antibody levels are strongly associated with recent *C. jejuni* infection in GBS patients. *C. jejuni* is a frequent antecedent pathogen associated with GBS in Bangladesh [6]. The association between anti-LOS and anti-ganglioside antibody reactivity is significant but not absolute. In some patients with anti-ganglioside antibodies we did not find a high IgG activity against LOS. This may result from relatively low titres or different fine-specificities of the anti-ganglioside antibodies in these patients. In our previous studies, we found no binding of anti-GM1 antibodies with LOS of *C. jejuni* from MFS and GBS patients [16]. There are some GBS patients without anti-ganglioside antibodies but with anti-LOS antibodies. Patients with a *C. jejuni* infection may have antibodies against complexes of gangliosides [17]. It is interesting to note that our preliminary data showed that patients with anti-LOS reactivity but without reactivity against single gangliosides can have antibodies against complexes gangliosides (data not shown). Our results are in agreement with recent observations that ganglioside complexes are important target antigens in GBS as well as in MFS [28], [29], [30].

Monoclonal antibodies against gangliosides were bound by LOS BD-07 and LOS BD-39 in a similar pattern. Serum DK-07 and DK-39 showed a dramatic reduction in anti- ganglioside reactivity after incubation with the autologous LOSs. Incubation with LOS that lacked ganglioside-mimics did not inhibit ganglioside-reactivity, demonstrating that serum DK-07 and serum DK-39 were specifically deprived of their ganglioside antibodies by the incubation with the autologous LOSs. This finding indicates that in these two GBS patients the antibodies against GM1 and GD1a were induced by LOS during the preceding *C. jejuni* infection. Immune response against *C. jejuni* is involved in the pathogenesis of GBS by cross-reactivity with neural tissue [17].

Various ganglioside mimics were found in the LOS of neuropathy-associated strains in Bangladesh. Carbohydrate moieties present in GM1 and GD1a were the most prevalent ganglioside mimic in GBS-associated *C. jejuni* strains, and it was predominantly found in LOS class A strains. This finding is consistent with the results previously described [12], [31], but in contrast with those of Nachamkin et al. who reported previously that the expression of GD1a, and not GM1 is associated with GBS [32]. Mass spectrometry analysis has not demonstrated authentic GQ1b-like structures in *C. jejuni* LOS. The detection of structures with a terminal di-NeuAc-Gal in both strains (BD-10 and BD-67) from Bangladesh associated with ophthalmoplegia suggests that in these patients, pathogenic antibodies are raised against GA2- and GD3-like LOS. Both strains express the same LOS outer core, which appears to be a novel structure with two extension sites from the inner core and two branches mimicking GA2 and GD3, respectively. There are alternative explanations for the observation that one GBS-associated strain (BD-94) did not express ganglioside mimic in its LOS. It has been demonstrated previously that GBS patients can occasionally be infected with two different *C. jejuni* strains. In such cases only one of the strains could be linked to GBS [12].

GBS is a clinically heterogeneous disorder, in which the neurological deficits are partly related to the specificity of the anti-ganglioside antibodies. In patients with preceding *C. jejuni* infections, the specificity of these cross-reactive antibodies is determined by the carbohydrate outer core of the *C. jejuni* LOS, which is controlled by genetic polymorphisms. [26], [33], [34]. The presence of, and polymorphism within the *cstII* gene in *C. jejuni* has been associated with both the expression of ganglioside mimics and the clinical features of patients with GBS [35], [36]. The Thr51 variant was associated with monosialylated LOS and seemed to occur more frequently in class A strains and in GBS-related strains (BD-07 and BD-39). We found that the *CstII* Asn51 variant was associated with the expression of disialylated LOS and seemed to occur more frequently in class B strains, and strains related with clinical symptoms of MFS or GBS with ophthalmoplegia.

Patients DK-07 and DK-39 have developed interesting immune responses. Bacterial genotypying showed that these patients were infected with clonal *C. jejuni* strains [37]. Mass spectrometry results and our LOS characterization data demonstrated that LOS BD-07 and BD-39 share the same ganglioside mimics. However, patient DK-07 developed an anti-GD1a immune response while patient DK-39 developed an anti-GM1 immune response. These results suggest that the immune response against gangliosides is not only determined by ganglioside mimicry present in the *C. jejuni* LOS but may also be determined by host genetic factors.

In conclusion, our study further supports the hypothesis that infections with specific *C. jeuni* strains induce cross-reactive IgG antibodies against LOS and gangliosides in GBS patients. The conformation of the oligosaccharide moieties in *C. jejuni* LOS and the induction of anti-ganglioside antibodies by the adaptive immune system cannot be predicted by the biochemical structure only. Further research is necessary to elucidate the mechanism by which *C. jejuni* determines the fine-specificity of the anti-ganglioside antibodies.

## Supporting Information

Figure S1 **Serum antibodies to ganglioside cross-reacted with LOS from the autologous** ***C. jejuni*** **strains (circles) but not with LOS from the control Penner HS:03 serostrain (CCUG 10937) lacking ganglioside mimicry (diamonds).** **A**) Inhibition of IgG anti-GD1a reactivity in serum from patient DK-07 by pre-incubation with LOS from the autologous *C. jejuni* BD-07 strain and from Penner HS:03 serostrain (CCUG 10937). **B**) Inhibition of IgG anti-GM1 reactivity in serum from patient DK-39 by pre-incubation with LOS from the autologous *C. jejuni* BD-39 strain and from Penner HS:03 serostrain (CCUG 10937).
(TIF)

Table S1 **Negative ion ESI-MS data and proposed compositions for** ***O*** **-deacylated LOS from** ***Campylobacter jejuni*** **BD-07, BD-10, BD-39, BD-67 and BD-94.**
(DOC)
