# Supplementary figures and images for: Guillain-Barré Syndrome-Related Campylobacter jejuni in Bangladesh: Ganglioside Mimicry and Cross-Reactive Antibodies
Source: PLoS One. 2012 Aug 27;7(8):e43976. doi: 10.1371/journal.pone.0043976 (PMC3428305; doi:10.1371/journal.pone.0043976)

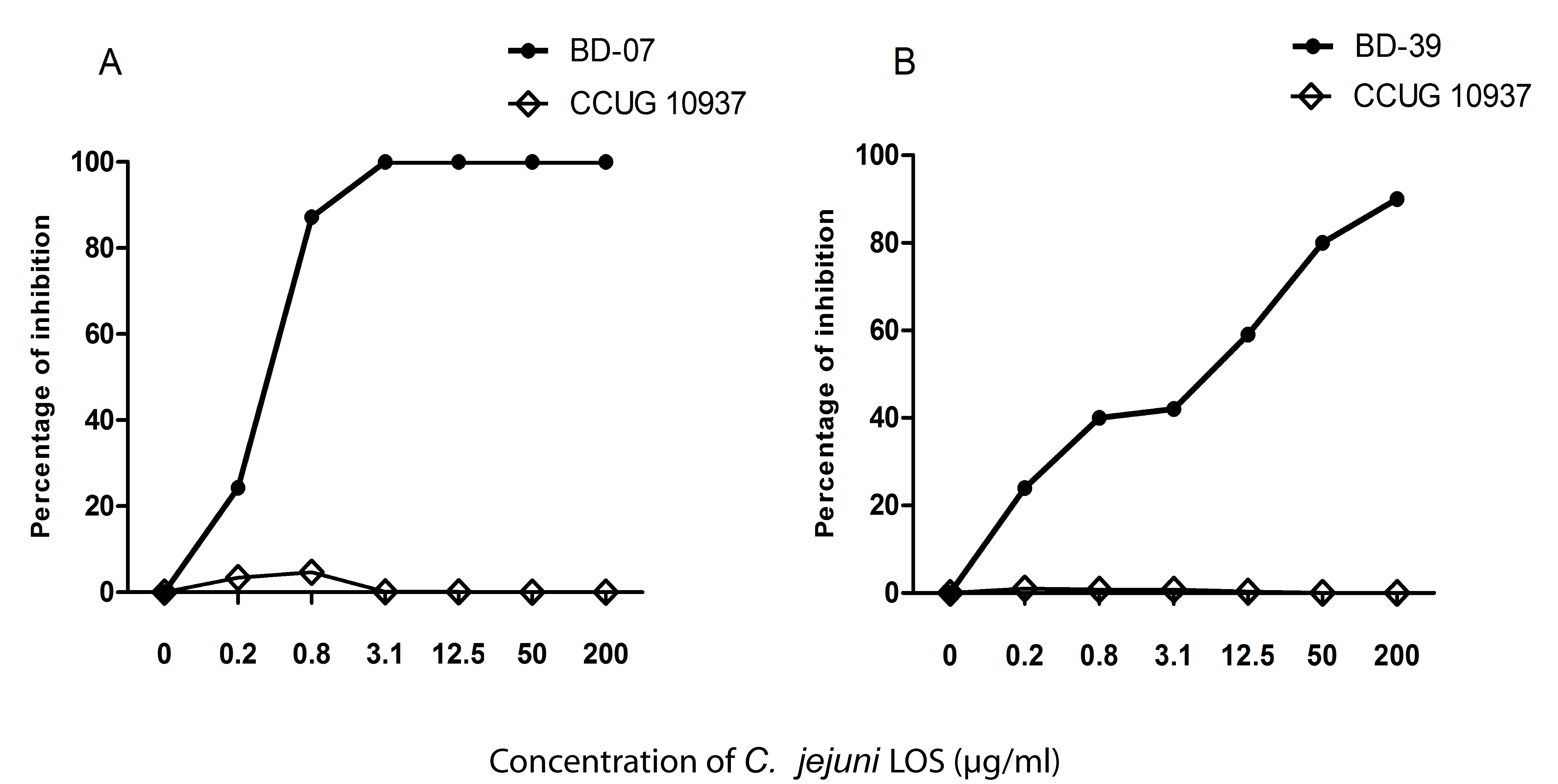

Supplement: Figure S1 — Serum antibodies to ganglioside cross-reacted with LOS from the autologous C. jejuni strains (circles) but not with LOS from the control Penner HS:03 serostrain (CCUG 10937) lacking ganglioside mimicry (diamonds). A) Inhibition of IgG anti-GD1a reactivity in serum from patient DK-07 by pre-incubation with LOS from the autologous C. jejuni BD-07 strain and from Penner HS:03 serostrain (CCUG 10937). B) Inhibition of IgG anti-GM1 reactivity in serum from patient DK-39 by pre-incubation with LOS from the autologous C. jejuni BD-39 strain and from Penner HS:03 serostrain (CCUG 10937). (TIF) [file pone.0043976.s001.tif]
