# Supplementary material for: Guillain-Barré Syndrome-Related Campylobacter jejuni in Bangladesh: Ganglioside Mimicry and Cross-Reactive Antibodies
Source: PLoS One. 2012 Aug 27;7(8):e43976. doi: 10.1371/journal.pone.0043976 (PMC3428305; doi:10.1371/journal.pone.0043976)
Supplement: Table S1 — Negative ion ESI-MS data and proposed compositions for O -deacylated LOS from Campylobacter jejuni BD-07, BD-10, BD-39, BD-67 and BD-94. (DOC) [file pone.0043976.s002.doc]

**Table S1.** Negative ion ESI-MS data and proposed compositions for *O*-deacylated LOS from *Campylobacter jejuni* BD-07, BD-10, BD-39, BD-67 and BD-94.

| **Strain** | **Observed ions**  **(*m/z*)** | | | | **Molecular mass**  **(Da)** | | **Proposed compositions** | | |
| --- | --- | --- | --- | --- | --- | --- | --- | --- | --- |
|  | **[M-4H]^4-^** | **[M-3H]^3-^** | **[M-2H]^2-^** | **Observed** | | **Calculated ^1^** | **Core oligosaccharide** | **Phosphorylation in lipid A** | **Acylation in lipid A** |
|  | 806.8 | 1075.9 |  | 3231.0 | | 3230.1 | Neu5Ac_1_•Hex_3_•HexNAc_1_•Hep_2_•*P*Etn_1_•KDO_2_ | *PP*Etn, *P* | 3 *N*-(3-OH 14:0) |
| **BD-07** | 837.6 | 1117.1 |  | 3354.8 | | 3353.1 | Neu5Ac_1_•Hex_3_•HexNAc_1_•Hep_2_•*P*Etn_1_•KDO_2_ | *PP*Etn, *PP*Etn | 3 *N*-(3-OH 14:0) |
|  | 894.0 | 1192.0 |  | 3579.5 | | 3578.5 | Neu5Ac_1_•Hex_3_•HexNAc_1_•Hep_2_•*P*Etn_1_•KDO_2_ | *PP*Etn, *PP*Etn | 4 *N*-(3-OH 14:0) |
|  | 966.8 | 1289.3 |  | 3871.1 | | 3870.7 | Neu5Ac_2_•Hex_3_•HexNAc_1_•Hep_2_•*P*Etn_1_•KDO_2_ | *PP*Etn, *PP*Etn | 4 *N*-(3-OH 14:0) |
|  |  | 979.1 | 1468.7 | 2939.9 | | 2938.8 | Hex_3_•HexNAc_1_•Hep_2_•*P*Etn_1_•KDO_2_ | *PP*Etn, *P* | 3 *N*-(3-OH 14:0) |
|  |  | 1020.0 | 1530.1 | 3062.6 | | 3061.9 | Hex_3_•HexNAc_1_•Hep_2_•*P*Etn_1_•KDO_2_ | *PP*Etn, *PP*Etn | 3 *N*-(3-OH 14:0) |
|  |  | 1054.1 | 1581.1 | 3164.8 | | 3164.2 | Hex_3_•HexNAc_1_•Hep_2_•*P*Etn_1_•KDO_2_ | *PP*Etn, *P* | 4 *N*-(3-OH 14:0) |
| **BD-10** | 829.0 | 1105.4 |  | 3319.6 | | 3318.1 | Neu5Ac_2_•Hex_3_•Hep_2_•*P*Etn_1_•KDO_2_ | *PP*Etn, *P* | 3 *N*-(3-OH 14:0) |
|  | 859.8 | 1146.7 |  | 3443.2 | | 3441.2 | Neu5Ac_2_•Hex_3_•Hep_2_•*P*Etn_1_•KDO_2_ | *PP*Etn, *PP*Etn | 3 *N*-(3-OH 14:0) |
|  | 885.4 | 1180.7 |  | 3545.4 | | 3544.5 | Neu5Ac_2_•Hex_3_•Hep_2_•*P*Etn_1_•KDO_2_ | *PP*Etn, *P* | 4 *N*-(3-OH 14:0) |
|  | 920.4 | 1227.4 |  | 3685.4 | | 3683.5 | Neu5Ac_2_•Hex_4_•HexNAc_1_•Hep_2_•*P*Etn_1_•KDO_2_ | *PP*Etn, *P* | 3 *N*-(3-OH 14:0) |
|  | 951.3 | 1268.4 |  | 3808.7 | | 3806.5 | Neu5Ac_2_•Hex_4_•HexNAc_1_•Hep_2_•*P*Etn_1_•KDO_2_ | *PP*Etn, *PP*Etn | 3 *N*-(3-OH 14:0) |
|  | 976.8 | 1302.3 |  | 3910.6 | | 3909.8 | Neu5Ac_2_•Hex_4_•HexNAc_1_•Hep_2_•*P*Etn_1_•KDO_2_ | *PP*Etn, *P* | 4 *N*-(3-OH 14:0) |
|  | 1007.5 | 1343.3 |  | 4033.5 | | 4032.9 | Neu5Ac_2_•Hex_4_•HexNAc_1_•Hep_2_•*P*Etn_1_•KDO_2_ | *PP*Etn, *PP*Etn | 4 *N*-(3-OH 14:0) |
|  | 806.9 | 1076.0 |  | 3231.3 | | 3230.1 | Neu5Ac_1_•Hex_3_•HexNAc_1_•Hep_2_•*P*Etn_1_•KDO_2_ | *PP*Etn, *P* | 3 *N*-(3-OH 14:0) |
| **BD-39** | 837.6 | 1117.1 |  | 3354.8 | | 3353.1 | Neu5Ac_1_•Hex_3_•HexNAc_1_•Hep_2_•*P*Etn_1_•KDO_2_ | *PP*Etn, *PP*Etn | 3 *N*-(3-OH 14:0) |
|  | 893.9 | 1192.2 |  | 3579.6 | | 3578.5 | Neu5Ac_1_•Hex_3_•HexNAc_1_•Hep_2_•*P*Etn_1_•KDO_2_ | *PP*Etn, *PP*Etn | 4 *N*-(3-OH 14:0) |
|  | 966.8 | 1289.3 |  | 3871.1 | | 3870.7 | Neu5Ac_2_•Hex_3_•HexNAc_1_•Hep_2_•*P*Etn_1_•KDO_2_ | *PP*Etn, *PP*Etn | 4 *N*-(3-OH 14:0) |
|  |  | 978.8 | 1468.5 | 2939.6 | | 2938.8 | Hex_3_•HexNAc_1_•Hep_2_•*P*Etn_1_•KDO_2_ | *PP*Etn, *P* | 3 *N*-(3-OH 14:0) |
|  |  | 1019.8 | 1530.0 | 3062.3 | | 3061.9 | Hex_3_•HexNAc_1_•Hep_2_•*P*Etn_1_•KDO_2_ | *PP*Etn, *PP*Etn | 3 *N*-(3-OH 14:0) |
|  |  | 1053.9 | 1581.0 | 3164.4 | | 3164.2 | Hex_3_•HexNAc_1_•Hep_2_•*P*Etn_1_•KDO_2_ | *PP*Etn, *P* | 4 *N*-(3-OH 14:0) |
| **BD-67** | 828.9 | 1105.6 |  | 3319.4 | | 3318.1 | Neu5Ac_2_•Hex_3_•Hep_2_•*P*Etn_1_•KDO_2_ | *PP*Etn, *P* | 3 *N*-(3-OH 14:0) |
|  | 859.7 | 1146.5 |  | 3442.9 | | 3441.2 | Neu5Ac_2_•Hex_3_•Hep_2_•*P*Etn_1_•KDO_2_ | *PP*Etn, *PP*Etn | 3 *N*-(3-OH 14:0) |
|  | 885.4 | 1180.7 |  | 3545.2 | | 3544.5 | Neu5Ac_2_•Hex_3_•Hep_2_•*P*Etn_1_•KDO_2_ | *PP*Etn, *P* | 4 *N*-(3-OH 14:0) |
|  | 920.2 | 1227.2 |  | 3684.7 | | 3683.5 | Neu5Ac_2_•Hex_4_•HexNAc_1_•Hep_2_•*P*Etn_1_•KDO_2_ | *PP*Etn, *P* | 3 *N*-(3-OH 14:0) |
|  | 950.9 | 1268.3 |  | 3807.8 | | 3806.5 | Neu5Ac_2_•Hex_4_•HexNAc_1_•Hep_2_•*P*Etn_1_•KDO_2_ | *PP*Etn, *PP*Etn | 3 *N*-(3-OH 14:0) |
|  | 976.6 | 1302.1 |  | 3909.9 | | 3909.8 | Neu5Ac_2_•Hex_4_•HexNAc_1_•Hep_2_•*P*Etn_1_•KDO_2_ | *PP*Etn, *P* | 4 *N*-(3-OH 14:0) |
|  | 1007.5 | 1343.1 |  | 4033.2 | | 4032.9 | Neu5Ac_2_•Hex_4_•HexNAc_1_•Hep_2_•*P*Etn_1_•KDO_2_ | *PP*Etn, *PP*Etn | 4 *N*-(3-OH 14:0) |
| **BD-94** | 918.8 |  |  | 3979.2 | |  | Unknown |  |  |
|  | 975.1 | 1300.1 |  | 3903.9 | |  | Unknown |  |  |

Isotope-average mass units were used for calculation of molecular mass values based on proposed compositions as follows: Hex,162.14; HexNAc, 203.19; Hep, 192.17; KDO, 220.18; *P*, 79.98; *P*Etn, 123.05; Neu5Ac, 291.26; HexN, 161.16; HexN3N, 160.18; C14:0 3-OH, 226.36; H_2_O, 18.01.
